# Estimation of case-fatality rate in COVID-19 patients with hypertension and diabetes mellitus in the New York state: a preliminary report

**DOI:** 10.1017/S0950268821000066

**Published:** 2021-01-08

**Authors:** Yang Ge, Shengzhi Sun, Ye Shen

**Affiliations:** 1 Department of Epidemiology and Biostatistics, The University of Georgia, Athens, GA, USA; 2 Department of Environmental Health, Boston University School of Public Health, Boston, MA, USA

**Keywords:** COVID-19, diabetes, hypertension

## Abstract

Pre-existing health conditions may exacerbate the severity of coronavirus disease 2019 (COVID-19). We aimed to estimate the case-fatality rate (CFR) and rate ratios (RR) for patients with hypertension (HBP) and diabetes mellitus (DM) in the New York state. We obtained the age-specific number of COVID-19 confirmed cases and deaths from public reports provided by the New York State Department of Health, and age-specific prevalence of HBP and DM from the Behavioral Risk Factor Surveillance System 2017. We calculated CFR and RR for COVID-19 patients with HBP and DM based on the reported number of deaths with the comorbidity divided by the expected number of COVID-19 cases with the comorbidity. We performed subgroup analysis by age and calculated the CFR and RR for ages of 18–44, 45–64 and 65+ years, respectively. We found that the older population had a higher CFR, but the elevated RRs associated with comorbidities are more pronounced among the younger population. Our findings suggest that besides the elderly, the young population with comorbidity should also be considered as a vulnerable group.

As of 24 August 2020, coronavirus disease 2019 (COVID-19) has widely affected countries and regions [1]. The New York state was heavily impacted by the coronavirus with more than 22 thousands of deaths [2]. Prior studies suggest that pre-existing health conditions such as hypertension (HBP) and diabetes mellitus (DM) may exacerbate the severity of COVID-19 [3, 4]. However, few studies have estimated the corresponding case-fatality rate (CFR) for patients with these two comorbidities. The timely analysis is highly needed in emerging disease outbreaks but is commonly hindered by the time-consuming process of data collection and cleaning.

In this study, we aim to calculate the CFR for adults with COVID-19 and with HBP (CFR_HBP_) and DM (CFR_DM_) using aggregated level data from multiple sources. Quantitative assessments of the risk of death associated with them can inform disease control globally.

We first obtained the total number of COVID-19 cases and deaths provided by the New York State Department of Health [2]. We then calculated the expected number of confirmed cases with comorbidities by the prevalence reported from the Behavioral Risk Factor Surveillance System 2017 (BRFSS) [5]. We eventually calculated the CFR_HBP_ or CFR_DM_ as the total number of deaths with the corresponding comorbidity divided by the expected total number of COVID-19 cases with that comorbidity. To further control for age, we performed stratified analysis and calculated the CFR by different age groups of 18–44, 45–64 and 65+ years.

The New York State Department of Health provides daily counts of confirmed cases from all lab testing samples and deaths from health care facilities in the New York state in the New York State Department of Health COVID-19 Tracker [2] (titled as ‘TESTING AND POSITIVE CASES’ on the website). The tracker also provides age-group specific numbers of COVID-19 deaths with comorbidities of HBP (D_HBP_) or DM (D_DM_).

Note that the counts of confirmed cases were reported without age group information in the New York state. We estimated the age group-specific number of cases according to the age distribution of confirmed COVID-19 cases in the contiguous US reported from the *Centers for Disease Control and Prevention* [6]. We also do not have information on comorbidities for counts of confirmed cases. To estimate the number of cases with HBP (C_HBP_) or DM (C_DM_), we multiplied the counts of confirmed cases by the corresponding prevalence of HBP and DM in the New York state from BRFSS [5] 2017, assuming that the susceptibility of COVID-19 infection for patients of these two comorbidities is similar to the general population. More details are provided in the supplementary material.

We then calculated the CFR for HBP (CFR_HBP_) or DM (CFR_DM_) overall and by age group (18–44, 45–64 and 65+ years). We further obtained the CFR ratio (RR) to compare the risk of COVID-19 death with and without the comorbidities.

We included a total of 340 338 (96.9% of all cases) confirmed cases and 22 708 (99.9% of all deaths) deaths by 18 May in the New York state. HBP and DM in the New York state were two common comorbidities of COVID-19 cases with the estimated prevalence of 32.2% (95% CI: 30.4%, 34.0%) and 11.9% (95% CI: 10.6%, 13.2%), respectively. As expected, the prevalence of HBP (Fig. 1a) and DM (Fig. 1b) was highest among patients aged 65 years and above, with the CFR being 21.08% (95% CI: 20.25%, 21.99%) for HBP (Fig. 1c) and 30.53% (95% CI: 28.11%, 33.40%) for DM (Fig. 1d). Compared with COVID-19 patients who are free of HBP, those with HBP are associated with higher CFR ratios of 2.6 (95% CI: 2.3, 2.9), 1.7 (95% CI: 1.5, 1.8) and 1.0 (95% CI: 0.9, 1.1) for patients aged 18–44, 45–64 and 65+ years, respectively. The corresponding CFR ratios for DM were 15.2 (95% CI: 12.2, 19.9), 4.1 (95% CI: 3.6, 4.6) and 1.7 (95% CI: 1.5, 1.9), respectively (Fig. 1e).

**Fig. 1.**
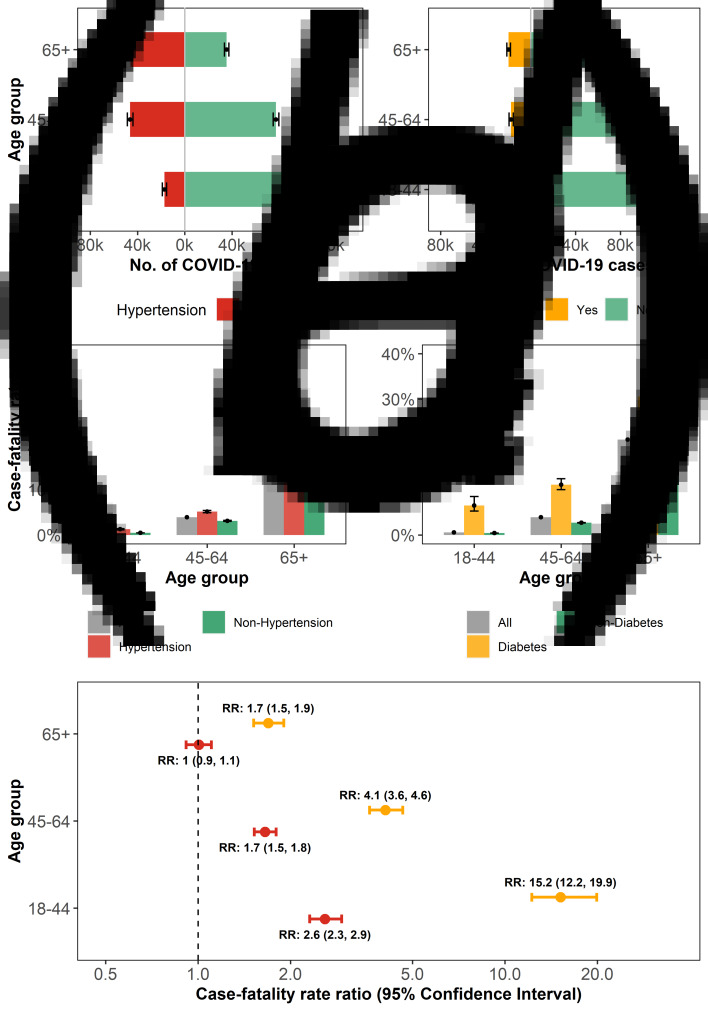
Estimation of the CFR in COVID-19 patients with HBP or DM. (**a**): Age distribution of confirmed COVID-19 cases with HBP. (**b**): Age distribution of confirmed COVID-19 cases with DM. (**c**): Age group-specific CFR among COVID-19 patients in all cases, with or without HBP, respectively. (**d**): The COVID-19 age group-specific CFR in all cases, with or without DM, respectively. (**e**): The age group-specific CFR ratios (RR) of COVID-19 cases with HBP or DM.

We found that COVID-19 patients with HBP and DM were associated with an increased risk of death in the New York state. While the elderly population had a higher CFR, the elevated CFR ratios associated with comorbidities are more pronounced for the younger population. Higher risks of COVID-19 severity being associated with comorbidities were reported in some countries [7, 8] but less common in regions like sub-Saharan Africa. Several hypotheses were proposed to explain the geographic variations in COVID-19 risk. The genetic difference of angiotensin converting enzyme-2 (ACE2) may exist between different ethnicities [9] with assumptions that African Americans might be less susceptible to COVID-19. In addition, various common medicines [10] currently used in certain areas, such as hydroxychloroquine [11] could have biased the association between comorbidities and COVID-19. These findings suggest that the likelihood of getting an infection and being severe after infection should be studied separately. Our study suggested that physicians should consider young COVID-19 cases who have comorbidities as a high-risk group. Additional attentions should also be given to this group in the discussion of non-pharmaceutical interventions, treatments and vaccine deployments.

In this study, we provided a framework to estimate the severity of COVID-19 for patients with comorbidities using aggregated data from multiple sources. Our approach is useful especially when individual-level data are unavailable for an extensive period of time, which is common in the early stage of a pandemic. Also, we used the surveillance data from an entire state or country. Findings from our study are more likely to be generalisable to the overall population infected by COVID-19. Our results should be considered as preliminary estimations with several limitations. First, we do not have age-specific numbers of confirmed cases with comorbidities. To calculate age group-specific results, we relied on information from data sources of CDC and BRFSS. Therefore, if HBP or DM causes a significant increase in COVID-19 susceptibility, our estimation may underestimate the total number of COVID-19 cases with these comorbidities, and subsequently lead to higher estimations of CFR_HBP_ or CFR_DM_. Second, due to data availability, we used the most recent prevalence of HBP and DM in BRFSS (2017), which might introduce some degrees of bias. However, the prevalence of HBP and DM is relatively stable, thus the bias should be minimum. Further studies with detailed individual data are warranted to evaluate our estimation.

## Data Availability

The data that support the findings of this study are openly available in COVID-19 TRACKER at https://coronavirus.health.ny.gov/home.
